# Epistasis of polymorphisms related to the articular cartilage extracellular matrix in knee osteoarthritis: Analysis-based multifactor dimensionality reduction

**DOI:** 10.1590/1678-4685-GMB-2018-0349

**Published:** 2020-03-27

**Authors:** Javier Fernández-Torres, Gabriela Angélica Martínez-Nava, Yessica Zamudio-Cuevas, Carlos Lozada, Daniela Garrido-Rodríguez, Karina Martínez-Flores

**Affiliations:** 1 Synovial Fluid Laboratory, Instituto Nacional de Rehabilitación “Luis Guillermo Ibarra Ibarra”, Mexico City, Mexico.; 2 Rheumatology Service, Instituto Nacional de Rehabilitación “Luis Guillermo Ibarra Ibarra”, Mexico City, Mexico.; 3 Center for Research in Infectious Diseases, National Institute of Respiratory Diseases, Mexico City, Mexico.

**Keywords:** Epistasis, extracellular matrix, knee osteoarthritis, multifactor dimensionality reduction, polymorphisms

## Abstract

Osteoarthritis (OA) is a complex disease with a multifactorial etiology. The genetic component is one of the main associated factors, resulting from interactions between genes and environmental factors. The aim of this study was to identify gene-gene interactions (epistasis) of the articular cartilage extracellular matrix (ECM) in knee OA. Ninety-two knee OA patients and 147 healthy individuals were included. Participants were genotyped in order to evaluate nine variants of eight genes associated with ECM metabolism using the OpenArray technology. Epistasis was analyzed using the multifactor dimensionality reduction (MDR) method. The MDR analysis showed significant gene-gene interactions between *MMP3* (rs679620) and *COL3A1* (rs1800255), and between *COL3A1* (rs1800255) and *VEGFA* (rs699947) polymorphisms, with information gain values of 3.21% and 2.34%, respectively. Furthermore, in our study we found interactions in high-risk genotypes of the *HIF1AN*, *MMP3* and *COL3A1* genes; the most representative were [AA+CC+GA], [AA+CT+GA] and [AA+CT+GG], respectively; and low-risk genotypes [AA+CC+GG], [GG+TT+GA] and [AA+TT+GA], respectively. Knowing the interactions of these polymorphisms involved in articular cartilage ECM metabolism could provide a new tool to identify individuals at high risk of developing knee OA.

## Introduction

To date, knee osteoarthritis (OA) prevails as the main cause of physical disability in senior adults. It is well known that age, gender, overweight, and genetic predisposition are key factors for its development (Arden and Nevitt*,* 2006; Peláez-Ballestas *et al.*, 2011; Hernández-Cáceres *et al.*, 2015). Articular cartilage, synovial membrane, and subchondral bone are tissues affected by OA; however, other tissues, such as meniscus and ligaments are compromised too. Progressive degeneration of articular cartilage, the subsequent decrease in joint space and osteophyte formation, as well as proteoglycans and collagen loss, extracellular matrix (ECM) mineralization, and hypertrophic differentiation of chondrocytes represent the hallmark of OA (De Filippis *et al.*, 2004; Bertrand *et al.*, 2010; Kraus *et al.*, 2015).

Healthy articular cartilage is a hyaline, viscoelastic, avascular, aneural, and alymphatic tissue. It is composed of an ECM rich in collagens (types II, IX and XI), proteoglycans (aggrecan), and water. The cellular part consists of chondrocytes only (Sophia *et al.*, 2009). As a result of being an avascular tissue, oxygen and nutrients are lower than in other tissues, creating a hypoxic environment (0.5 – 10% of oxygen, or 4 – 70 mm Hg, respectively, which also depends of the zone in tissue, either deep or superficial) (Pfander *et al.*, 2006; Oswald *et al.*, 2008; Ströbel *et al.*, 2010). Despite the extreme conditions of the cartilage, hypoxia is necessary in metabolic processes, such as chondrogenesis ECM synthesis and degradation. It also modulates the subchondral bone angiogenesis through the vascular endothelial growth factor A (VEGFA). These processes, and the proper maintenance of general cartilage homeostasis, are coordinated by hypoxia-inducible factor-1α (HIF-1α) (Pfander *et al.*, 2006). If the ECM synthesis process is decreased, cartilage will become thinner and weaker, whereas it will turn hypertrophic and disorganized with a ECM synthesis increase, as a way to compensate for the null ECM generated favoring osteophyte formation (Martel-Pelletier *et al.*, 2008). In advanced life stages, or during the development of joint diseases, such as OA, the delicate balance between ECM synthesis and degradation is heavily affected by factors responsible for articular cartilage degradation, among which stand out the proinflammatory cytokines (IL-1β, IL-6, IL-8, TNF-α), chemokines, metalloproteinases (MMP-1, 3, 9 and 13), and aggrecanases (ADAMTS 4, 5 and 9) (Mariani *et al.*, 2014). In addition to these molecules, EGFR ligands are able to stimulate the expression of genes related to cartilage catabolism and, on the other hand, inhibit the catabolic activity of chondrocytes (Long *et al.*, 2015). Although VEGFA plays a beneficial role for the articular cartilage, during OA it is actively involved in remodeling the ECM and forming osteophytes (Hamilton *et al.*, 2016). On the other hand, the concentration of nitric oxide (ON) generated by nitric oxide synthase (NOS) regulates MMP synthesis. At low concentrations it inhibits production, but at high concentrations, it activates them, favoring ECM degradation. It also promotes free radical production and induces chondrocyte apoptosis (Abramson, 2008a,b).

From a genetic standpoint, several studies suggest associations between single-nucleotide polymorphisms (SNP) and knee OA (Fernández-Moreno *et al.*, 2008; van Meurs and Uitterlinden, 2012; Fernández-Torres *et al.*, 2017; Ozcan *et al.*, 2017). Nevertheless, most of them were assessed individually, in contrast to joint assessments through gene-gene interactions (epistasis), which could provide more information regarding their role. Recently, Wang *et al.* (2016) published a meta-analysis associating different SNPs with knee, hip, and hand OA risk, and suggesting that genetic predisposition is a key factor for OA development. Another relevant aspect to be considered is the fact that SNP distribution in this kind of studies is greatly affected by ethnicity and geographic location.

Several studies related to the analysis of gene-gene interactions in complex diseases have only been conducted using logistic regression models, linkage disequilibrium, and Hardy-Weinberg equilibrium tests, all of which have limitations (Ritchie *et al.*, 2003; Mechanic *et al.*, 2008). Therefore, identification and characterization of gene-gene and gene-environment interactions have been limited primarily due to a lack of powerful statistical methods, and particularly because of small sample sizes, which has been a challenge for geneticists. In this sense, the multifactor dimensionality reduction (MDR) method does not require a model as such, given that no genetic models are assumed, neither is it parametric, as no parameters are estimated.

Dimensionality is defined as the interactions between genetic or environmental factors, which increase exponentially as these factors increase. MDR reduces the dimensionality of multilocus data to improve the ability to detect genetic combinations that confer disease risk. MDR pools genotypes into ‘high-risk’ and ‘low-risk’ or ‘response’ and ‘non-response’ groups in order to reduce multidimensional data into only one dimension (dichotomous variable). Because it is a non-parametric method, no hypothesis concerning the value of any statistical parameter is made. Additionally, MDR was designed to detect gene–gene or gene–environment interactions in datasets with categorical independent variables, such as SNP and other sequence variations (insertions, deletions, etc.), as well as environmental data that can be represented as categorical variables. The endpoint, or dependent variable, must be dichotomous, such as case/control for studies of human disease. Pharmacogenomics data can also be analyzed with MDR, in terms of ‘response/non-response’ or ‘toxicity/no toxicity’. MDR is appropriate for any data type with two distinct clinical endpoints (Motsinger and Ritchie, 2006; Moore and Andrews, 2015).

For studies with more than two factors, the steps of the MDR method are repeated for each possible model size (two-factor, three-factor, etc.), if computationally feasible. The result is a set of models, one for each model size considered. From this set, the model with the combination of loci and/or discrete environmental factors that maximize the cross validation consistency and minimize the prediction error is selected. Cross-validation consistency is a measure of the number of times an MDR model is identified in each possible 90 per cent of the subjects. When cross-validation consistency is maximal for one model and prediction error is minimal for another model, statistical parsimony is used to choose the best model. On the other hand, a balanced accuracy function is used when the ratio of cases to controls is different than one (Motsinger and Ritchie, 2006; Moore and Andrews, 2015).

The MDR method is based on an algorithm that generates the characteristics or attributes created by a new variable or attribute. It has also developed an entropy-based method for a better statistical interpretation of the results (Hahn *et al.*, 2003; Cho *et al.*, 2004; Gui *et al.*, 2011; Su *et al.*, 2015; Kohli *et al.*, 2016). Previously, Su *et al.* (2015) performed an epistasis study between TGF-β and Smad3 polymorphisms and OA patients, revealing that it can affect the development of OA. Recently, our work group published a study of epistasis between polymorphisms related to oxidative stress and OA patients, and the results have shown a strong interaction between *ADIPOQ* (rs1501299) and *PON1* (rs662) polymorphisms (Fernández-Torres *et al.*, 2019), suggesting an important role in the OA pathogenesis; both studies were carried out using the MDR method. The generalized MDR (GMDR) method is an extension from MDR. It allows an adjustment for discrete and quantitative covariables and can be applied to both dichotomous and continuous phenotypes in several study designs based on population. Additionally, a command sequence includes *p*-value assessment (Lou *et al.*, 2007; Chen *et al.*, 2011).

Interactions between multiple polymorphisms of different genes could be the foundation of the genetic origin of OA. Therefore, this study is focused on evaluating whether interactions between several genetic variants of articular cartilage ECM are associated with knee OA in a Mexican population.

## Subjects and Methods

### Subjects

Two hundred thirty-nine unrelated individuals of Mexican ancestry and geographically matched were included in this case-control study. Ninety-two of them were knee OA patients of both genders, recruited from the Rheumatology Service of the Instituto Nacional de Rehabilitación “Luis Guillermo Ibarra Ibarra” (INR-LGII). Knee OA was diagnosed under the guidelines of the American College of Rheumatology (Altman *et al.*, 1986); the clinical exam and the X-ray evaluation were performed by a rheumatologist and a radiologist, respectively. OA severity was evaluated using the Kellgren-Lawrence radiological scale (K-L) (Kellgren and Lawrence, 1957). Patients with K-L ≥ 2 were included, and those with other etiologies causing knee diseases, such as inflammatory arthritis (rheumatoid arthritis or any other autoimmune disease), post-traumatic arthritis, post-septic arthritis, poliomyelitis, or skeletal dysplasia, were excluded. One hundred forty-seven healthy employees of the INR-LGII of both genders, with no signs or symptoms of knee OA, OA family history, and no other type of arthritis or joint painful condition were recruited as controls from the departments of Human Communications and Human Resources, as well as the cleaning staff. They were invited to participate in the study through the proper authority. Additionally, all evaluated controls showed a K-L grade equal to or lower than one. All participants were at least 40 years old and signed an informed consent. Data on age, gender, body mass index (BMI), and birth place was collected through an interview. This study was conducted under the criteria set forth in the Declaration of Helsinki and was approved by the INR-LGII Ethics and Research Committee (CONBIOETICA-09CEI-031-20171207).

### SNPs selection and genotyping

For this study, nine candidate SNPs from eight genes involved in articular cartilage ECM were selected. The search strategy was as follows: a) information of each SNP was obtained from the public databases Hap Map (http://www.hapmap.ncbi.nlm.nih.gov/) and the National Center for Biotechnology Information dbSNP database (http://www.ncbi.nlm.nih.gov/snp); b) the previously selected SNPs evaluated in OA or other pathologies (Rodriguez-Lopez *et al.*, 2006; Jacobs *et al.*, 2008; Chen and Mattey, 2012; Zhan *et al.*, 2013; Gibbon *et al.*, 2017; Guo *et al.*, 2017; Kaynak *et al.*, 2017; Du *et al.*, 2019); and c) the selected polymorphisms should not be in linkage disequilibrium between them. Finally, polymorphisms with minor allele frequency (MAF) in the Mexican population <10% were excluded (Table 1). Selected SNPs that were genotyped in cases and controls were rs699947, rs3025039 (*VEGFA*), rs11292 (*HIF1AN*), rs1800255 (*COL3A1*), rs4444903 (*EGF*), rs679620 (*MMP3*), rs2252070 (*MMP13*), rs2297518 (*NOS2*), and rs2070744 (*NOS3*).

**Table 1:** Single-nucleotide polymorphisms (SNPs) studied.

Closest gene	db SNP rs ID	Chromosome: position	Location	MAF in Mexican population	Disease	OR*	*n*-size	Reference
*VEGFA*	rs699947	Chr.6:43736389	5’UTR	0.42 (A)	RA	-	413	Chen and Mattey, 2012
*VEGFA*	rs3025039	Chr.6:43752536	3’UTR	0.30 (T)	PC	0.91	1621	Jacobs *et al*., 2008
*HIF1AN*	rs11292	Chr.10:102313607	3’UTR	0.13 (G)	BC	1.12	1056	Du *et al*., 2019
*COL3A1*	rs1800255	Chr. 2:189864080	Exon	0.23 (A)	ACL	0.78	321	Stepien-Soldowska *et al.*, 2015
*EGF*	rs4444903	Chr.4:110834110	5’UTR	0.38 (A)	PC	0.98	1636	Jacobs *et al.*, 2008
*MMP3*	rs679620	Chr.11:102713620	Missense variant	0.31 (T)	OA	1.45	297	Guo *et al.*, 2017
*MMP13*	rs2252070	Chr.11:102826539	5’UTR	0.29 (C)	RA	1.13	1202	Rodriguez-Lopez *et al.*, 2006
*NOS2*	rs2297518	Chr.17:26096597	Missense variant	0.13 (A)	PC	1.03	1637	Jacobs *et al.*, 2008
*NOS3*	rs2070744	Chr.7:150690079	Intron	0.27 (C)	PC	0.90	1080	Jacobs *et al.*, 2008

MAF, minor allele frequency; ECM, extracellular matrix; *OR, corresponding to the minor homozygous genotype in a codominant model; *n*-size, cases + controls; OA, osteoarthritis; AR, rheumatoid arthritis; PC, prostate cancer; BC, breast cancer; ACL, anterior cruciate ligament. All studies were case-control design.

Given that the Mexican population structure is composed of a mix of Amerindian, European, and African ancestors, a set of nine ancestry-informative markers (AIMs) was used to evaluate whether any resulting association could be a confounder due to the population stratification during the data adjustment step (Bonilla *et al.*, 2004; Choundry *et al.*, 2006) (Table S1).

DNA was isolated from peripheral blood in EDTA tubes, using a commercial kit (QIAmp 96 DNA Blood Kit, Qiagen, Hilden, Germany). The samples were genotyped through the OpenArray technology on a QuantStudio 12K flex equipment (Thermo Fisher Scientific). Genomic DNA from the samples was adjusted to 50 ng/μL; 2.5 μL of DNA were mixed with 2.5 μL of TaqMan OpenArray Genotyping Master Mix (Thermo Fisher Scientific) in 384-well trays. The mixtures were loaded onto OpenArray genotyping trays using the AccuFill system (Thermo Fisher Scientific). Amplification was conducted following the manufacturer’s protocol. Raw results were analyzed with the QuantStudio 12K Flex software v1.2.2.

### Statistical analysis

The clinical variables were evaluated with Student’s *t*-test or Fisher’s exact test, when appropriate, and values were expressed as mean ± SD. Gene and allele frequencies of all polymorphisms were calculated and compared between cases and controls using Fisher’s exacts test. In order to control the global false positive rate, only SNPs with a statistically significant *p*-value in Fisher’s exact test were considered in the multivariate analysis. Associations of each SNP with OA risk were assessed with logistic regression models adjusted by age, gender, BMI, and ancestry, taking into account a codominant inheritance model for the SNP. The Bonferroni’s test was used to determine the significance level to correct multiple test errors, which taking into account the nine selected SNPs, an adjusted *p*-value < 0.005 (0.05/9) was considered statistically significant. Hardy-Weinberg equilibrium (HWE) in controls was calculated using the chi-squared test.

We used the STRUCTURE software v2.3.4 (Pritchard Lab, Stanford University, USA) to evaluate the effect of population stratification on the associations found of each population k (k = 3) with the genotypes of the nine AIMs mentioned above (Table S1). This information was included in the logistic regression models to adjust the associations found between the studied polymorphism and OA by individual mix.

The effect of the associated polymorphisms on groups stratified by gender and age (early OA, <50 years; late OA, >50 years) was analyzed (Table S2). All statistical analyses were performed with the STATA v14.0 (StataCorp, Texas, USA) statistical package considering an α = 0.05 significance level.

In order to study the epistasis, we used the MDR v3.0.2 or GMDR v0.9 statistical packages with Ritchie’s algorithm (Ritchie *et al.*, 2003). To corroborate the interaction model proposed by the MDR method, a logistic regression model was carried out, which included the SNPs suggested by the MDR. Subsequently, the *p*-value of the model, the adjusted r^2^, as well as the *p*-values for the ORs of the SNPs, were evaluated (Table S3).

## Results

### Characteristics of the study population

Table 2 shows the demographic and clinical characteristics of cases (n = 92) and controls (n = 147). Cases were significantly older than controls (47.2 ± 12.4 *vs.* 40.9 ± 12.0 years, *p* = 0.0001). Gender ratio was similar among the study groups, with women being predominant in both. BMI showed overweight in OA patients, whereas the control group showed normal weight, albeit on the upper threshold. The majority of cases and controls were from Mexico City (77.2% and 84.3%, respectively).

**Table 2:** Characteristics of the study population.

Parameter	Total (n=239)	OA (n=92)	Controls (n=147)	*p*
Age (years)	43.4 ± 12.5	47.2 ± 12.4	40.9 ± 12.0	**0.0001**
Gender				
Female (%)	185 (77.4)	80 (87.0)	105 (71.4)	0.005*
Male (%)	54 (22.6)	12 (13.0)	42 (28.6)	
BMI (Kg/cm^2^)	26.5 ± 4.76	29.0 ± 4.19	24.8 ± 4.38	**<0.0001**
Place of birth				
Mexico City	195 (81.5)	71 (77.2)	124 (84.3)	0.40*
Others states of Mexico (central region)	44 (18.5)	21 (22.8)	23 (15.7)	

Data are expressed as mean ± SD. *p*-values were estimated using Students *t*-test, α=0.05; p*-values were estimated using Fisher’s exact test, α=0.05. BMI, body-mass index, normal: 18.5 – 24.9; overweight: 25.0 – 29.9; obesity: ≥30.0. Significant *p*-values are in bold.

### Genetic and allelic frequencies of the SNPs studied in OA patients and controls

After adjusting for age, gender, BMI and ancestry, there were no significant differences between the genotype or allele frequencies of the analyzed polymorphisms in the study groups. Genotype distributions were compatible with HWE in controls, except for the *HIF1AN* (rs11292) polymorphism (Table 3).

**Table 3:** Genetic and allelic frequencies of SNPs studied in OA patients and controls.

Gene (SNP rs ID)	OA N (%)	Controls N (%)	OR*	(95% CI)	*p*	HWE in controls
*VEGFA* (rs3025039)						
CC	31 (38.7)	57 (47.9)	1.00	Reference		
CT	37 (46.2)	44 (37.0)	1.48	(0.76 – 2.88)	0.25	0.061
TT	12 (15.0)	18 (15.1)	1.23	(0.50 – 3.03)	0.64	
T	61 (38.1)	79 (33.6)	1.20	(0.77 – 1.88)	0.41	
*VEGFA* (rs699947)						
CC	39 (48.1)	57 (50.0)	1.00	Reference		0.063
CA	34 (42.0)	41 (36.0)	1.03	(0.50 – 2.12)	0.93	
AA	8 (9.90)	16 (14.0)	0.54	(0.17 – 11.7)	0.28	
A	50 (30.9)	73 (32.0)	0.81	(0.49 – 1.35)	0.43	
*HIF1AN* (rs11292)						
AA	61 (81.3)	51 (58.6)	1.00	Reference		0.011
AG	0 (0.00)	25 (28.7)	-	-	-	
GG	14 (18.7)	11 (12.6)	0.68	(0.25 – 1.78)	0.43	
G	28 (18.7)	47 (27.0)	0.58	(0.30 – 1.09)	0.09	
*COL3A1* (rs1800255)						
GG	35 (41.7)	30 (40.5)	1.00	Reference		0.102
GA	42 (50.0)	39 (52.7)	1.36	(0.63 – 2.92)	0.42	
AA	7 (8.33)	5 (6.76)	2.20	(0.41 – 11.7)	0.35	
G	112 (66.7)	99 (66.9)	1.00	Reference		
A	56 (33.3)	49 (33.1)	1.29	(0.76 – 2.20)	0.33	
*EGF* (rs4444903)						
GG	23 (28.0)	31 (27.9)	1.00	Reference		0.890
GA	41 (50.0)	56 (50.5)	1.04	(0.48 – 2.27)	0.90	
AA	18 (22.0)	24 (21.6)	1.51	(0.58 – 3.91)	0.39	
A	77 (47.0)	104 (46.8)	1.20	(0.75 – 1.92)	0.42	
*MMP3* (rs679620)						
CC	39 (48.7)	41 (51.9)	1.00	Reference		0.073
CT	30 (37.5)	27 (34.2)	2.17	(0.93 – 5.08)	0.07	
TT	11 (13.8)	11 (13.9)	0.88	(0.32 – 2.41)	0.81	
T	52 (32.5)	49 (31.0)	1.09	(0.64 – 1.87)	0.73	
*MMP13* (rs2252070)						
TT	38 (44.7)	46 (48.9)	1.00	Reference		0.602
TC	41 (48.2)	41 (43.6)	1.18	(0.62 – 2.26)	0.60	
CC	6 (7.10)	7 (7.50)	1.09	(0.32 – 3.71)	0.89	
C	53 (31.2)	55 (29.3)	1.09	(0.68 – 1.76)	0.70	
*NOS2* (rs2297518)						
GG	65 (81.2)	66 (80.5)	1.00	Reference		0.887
GA	14 (17.5)	15 (18.3)	0.86	(0.37 – 2.03)	0.75	
AA	1 (1.25)	1 (1.22)	1.51	(0.08 – 26.0)	0.78	
A	16 (10.0)	17 (10.4)	0.94	(0.44 – 2.01)	0.89	
*NOS3* (rs2070744)						
TT	58 (66.7)	53 (63.1)	1.00	Reference		0.092
TC	23 (26.4)	24 (28.6)	0.89	(0.42 – 1.86)	0.77	
CC	6 (6.90)	7 (8.33)	0.64	(0.19 – 2.17)	0.48	
C	35 (20.1)	38 (22.6)	0.80	(0.46 – 1.40)	0.45	

* The multi-variable model was adjusted for age (continuous data), gender (male, female), BMI (continous data) and admixture; OA, osteoarthritis patients; OR, Odds ratio; CI, confidence interval. HWE, Hardy-Weinberg equilibrium; if *p*<0.05, it is not consistent with HWE. Significant *p*-values are in bold.

### Polymorphisms distribution in groups stratified by gender and age groups

The polymorphism distributions stratified by gender and age are shown in Table S2. In men, the GA, CA, and GA heterozygote genotypes of the *COL3A1* (rs1800255), *VEGFA* (rs699947), and *EGF* (rs4444903) polymorphisms, respectively, were associated with a decreased OA risk (OR = 0.07, 95% CI = 0.00 – 0.84, *p* = 0.03; OR = 0.04, 95% CI = 0.00 – 0.85, *p* = 0.03; OR = 0.10, 95% CI = 0.01 – 0.94, *p* = 0.04, respectively). Regarding the analysis stratified by age groups, the CT heterozygote genotype of the *MMP3* (rs679620) polymorphism was associated with an increased risk of developing OA among the ≤ 50 year-old group (OR = 3.03, 95% CI = 1.07 – 8.93, *p* = 0.03), whereas the G minor allele of the *HIF1AN* (rs11292) polymorphism was associated with protection in the same age group (OR = 0.44, 95% CI = 0.22 – 0.88, *p* = 0.02). The AA homozygote genotype of the *VEGFA* (rs699947) polymorphism showed an association with protection for the over 50 year-old age group (OR = 0.06, 95% CI = 0.00 – 0.86, *p* = 0.03).

### MDR analysis

Table 4 summarizes the results of the MDR analysis, which assesses all possible combinations of the associated polymorphisms stratified by gender and age. According to the MDR analysis, the best model included the *MMP3* (rs679620), *COL3A1* (rs1800255), and *HIF1AN* (rs11292) polymorphisms. This model had a balanced accuracy test of 0.6434, a consistency of cross-validation of 10/10, and an interaction *p*-value = 0.0010. Additionally, *p*-values from logistic regression models were included.

**Table 4:** MDR analysis

*Locus* number	Model	Training Bal Acc	Testing Bal Acc	Cross-validation consistency	*p*	p*
1	HIF1AN_rs11292	0.5963	0.5391	6/10	0.3770	0.028
2	MMP3_rs679620, HIF1AN_rs11292	0.6390	0.6053	9/10	0.0547	0.085
3	MMP3_rs679620, COL3A1_rs1800255, HIF1AN_rs11292	0.6847	0.6434	10/10	**0.0010**	0.196
4	MMP3_rs679620, COL3A1_rs1800255, VEGFA_699947, HIF1AN_rs11292	0.7107	0.5614	7/10	0.1719	0.408
5	MMP3_rs679620, COL3A1_rs1800255, VEGFA_699947, HIF1AN_rs11292, EGF_rs4444903	0.7422	0.4674	10/10	0.8281	0.356

*p*-values were based on 1000 permutations and were obtained from sign test by MDR method.

*p-values were obtained from logistic regression models.

MDR, multifactor dimensionality reduction; Testing Bal Acc, testing-balanced accuracy.

Significant *p*-values are in bold.

**Figure 1:**
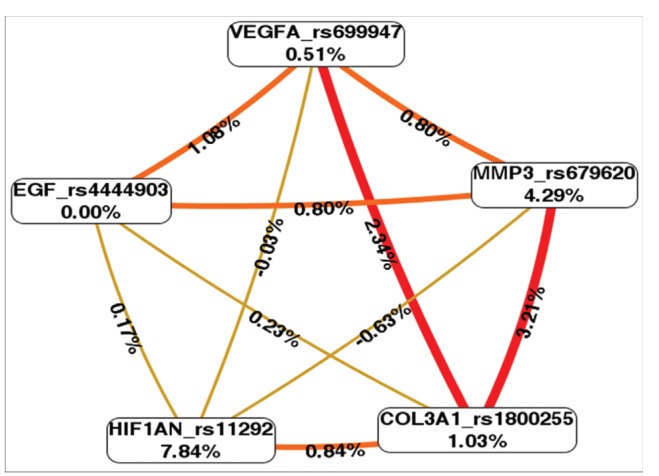
Interaction map for osteoarthritis risk. The interaction model describes the percentage of the entropy (information gain) that is explained by each factor or 2-way interaction. Values inside nodes indicate information gain of individual attributes or main effects, whereas values between nodes show information gain of pairwise combinations of attributes or interaction effects. Positive entropy (plotted in red or orange) indicates interaction, which can be interpreted as a synergistic or nonadditive relationship; while negative entropy (plotted in green-yellow) indicates independence or additivity (redundancy).

**Figure 2:**
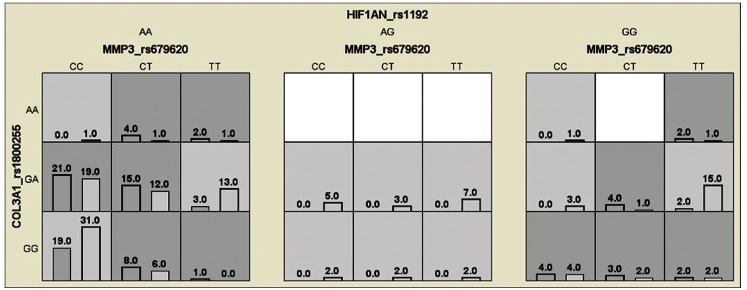
Distribution of high-risk and low-risk genotypes in the best three-locus model. The distribution shows high-risk (dark shading) and low-risk (light shading) genotypes associated with knee OA in the three-locus interaction detected by MDR analysis. The percentage of osteoarthritic subjects (left black bar in boxes) and control subjects (right hatched bar in boxes) is shown for each three-locus genotype combination. Boxes were labeled as high-risk if the ratio of the percentage of cases to controls met or exceeded the threshold of 1.0. Boxes were labeled as low-risk if the threshold was not exceeded. Based on the pattern of high-risk and low-risk genotypes, this three-locus model is evidence of gene to gene interaction.

Figure 1 shows the interaction map of the studied polymorphisms, based on entropy measures among individual variables. A strong interaction effect was observed between *MMP3* (rs679620) and *COL3A1* (rs1800255) polymorphisms, and between *COL3A1* (rs1800255) and *VEGFA* (rs699947) polymorphisms, with information gain values of 3.21% and 2.34%, respectively. Significance value was also confirmed through logistic regression models, with an interaction *p*-value = 0.036. Additionally, in our model we found interactions in high-risk genotypes of the *HIF1AN*, *MMP3* and *COL3A1* genes, the most representative were [AA+CC+GA], [AA+CT+GA] and [AA+CT+GG], respectively; and low-risk genotypes [AA+CC+GG], [GG+TT+GA] and [AA+TT+GA], respectively (Figure 2).

## Discussion

In this study, we applied the MDR computation method to assess the epistasis of candidate genes related to articular cartilage ECM in knee OA patients in a Mexican population. Our main findings revealed important gene-gene interactions between the *MMP3* (rs679620), *VEGFA* (rs699947), and *COL3A1* (rs1800255) polymorphisms.

The importance of analyzing MMP-3 (stromelysin-1) is that it is a zinc-dependent endopeptidase that catalytically degrades several collagen and non-collagen proteins of the basement membrane and the ECM. It is considered an essential regulator of ECM homeostasis in healthy and damaged musculoskeletal soft tissue (Gibbon *et al.*, 2017). The *MMP3* gene has several genetic polymorphisms that could considerably affect ECM degradation and remodeling. Cases and controls studies evaluating different SNPs within the *MMP3* gene have been conducted, including the rs679620 variant, yet none has been associated with OA risk (Guo *et al.*, 2017; Tong *et al.*, 2017). The non-association of this polymorphism that we observed in our work is consistent with previous reports, and it was unexpected, considering its functional role and the active involvement of this metalloprotease in ECM degradation in OA. This phenomenon has been reported in other pathologies, such as rheumatoid arthritis (Rodriguez-Lopez *et al.*, 2006). However, when we stratified by age group, we observed that carriers of the CT heterozygote genotype showed a significant association with OA risk in people younger than 50 years old, which might suggest the possibility that the polymorphism could be associated with a more aggressive disease phenotype or more rapid progression.

On the other hand, it has been proved that an increase in the VEGF protein level is associated with OA progression. Apparently, VEGF is involved in specific OA processes, including cartilage degeneration, osteophyte formation, subchondral bone cysts, sclerosis, synovitis, and pain. Moreover, inhibition of VEGF signaling decreases OA progression (Mariani *et al.*, 2014; Hamilton *et al.*, 2016). Genetically, there is little evidence of the association of *VEGFA* polymorphisms in OA pathogenesis. Sánchez-Enríquez *et al.* (2008) assessed the -460T/C (rs833061) and +405C/G (rs2010963) polymorphisms of *VEGFA* in knee arthrosis patients and compared them to healthy controls, but they did not find a significant association. In our study, we evaluated the rs699947 variant, and even though distribution was similar in cases and controls, we did find a genotype-phenotype association when stratifying by gender and age groups. We observed that the CA heterozygote genotype provides protection against OA development, but only among men, and that the AA homozygote genotype provides protection exclusively to individuals 50 years old or older. This variant is located in a promoter region of the *VEGFA* gene, but since its functional role in OA pathogenesis is not yet known, further research is needed.

Substitution of type-II collagen with type-III collagen in the articular cartilage ECM is a common feature of OA. Studies based on mRNA analysis prove that type-III collagen expression is associated with type-II collagen expression, which suggests a metabolic reaction of the chondrocytes to type-III collagen build-up in articular cartilage areas, probably as a response to mechanical injury or other damage to the ECM (Hosseininia *et al.*, 2016). To date, there is no scientific evidence of an association between polymorphisms of the *COL3A1* gene with knee OA. Stepien-Slodkowska *et al.* (2015) evaluated the rs1800255 variant among Polish patients with an anterior cruciate ligament injury and observed that the distribution of the AA homozygote genotype was higher in comparison to the control group (*p* = 0.0087). In our study, we evaluated the same variant among knee OA patients, and we found that the GA heterozygote genotype is significantly associated with protection, but just among men. This variant causes a change in Ala531Thr, but its role at the articular cartilage level is unknown. There might be a defect in procollagen III synthesis, resulting in a structural modification of the protein.

By using MDR analysis, we found evidence of two strong interactions with significant value between three out of nine variants of the eight candidate genes involved in ECM degradation that we assessed. Likewise, we could identify interactions between the rs11292 (*HIF1AN*), rs679620 (*MMP3*) and rs1800255 (*COL3A1*) polymorphisms, generating high and low risk haplotypes between OA patients. On the other hand, when we performed the interaction analysis using logistic regression models, we did not detect any interaction. One of the virtues of the MDR method is that it does not require very large sample sizes to detect interactions. However, logistic regression models require a very large “N” to detect statistical significance. Also, polymorphisms that are poorly represented or of low frequency are difficult to detect, and interactions between the polymorphisms can only be evaluated by pairs.

It is well known that OA pathogenesis is multifactorial, and its complexity is primarily due to its polygenic nature. Given this polygenic nature, it has been difficult to prove gene-gene interactions associated with knee OA. To date, only four studies have reported gene-gene interactions in knee OA susceptibility. The first of these studies evaluated the TGF-β/Smad3 signaling pathway using the MDR method (Su *et al.*, 2015). Other two focused on the Wnt/β-catenin signaling pathway and on the uric acid transporters polymorphisms, using logistic regression models (Fernández-Torres *et al.*, 2018a,b). The most recent one published by our working group, identified interactions between polymorphisms of the *ADIPOQ* and *PON1* genes, using the MDR method (Fernández-Torres *et al.*, 2019). The effect magnitude of any polymorphism is likely to be overlooked if the genes are individually examined, without considering potential interactions with other genes, especially those in related pathways.

Despite the results and the advantages offered by the MDR method, it is important to emphasize some aspects. Unlike classical genetic analysis, our main approach highlights the importance of evaluating in an integral manner the effect of genetic variants in knee OA. However, we are aware of certain limitations of our study, the first being that our sample size is limited. However, we believe that after performing a multivariate analysis and a rigorous selection of our patients and controls, the presented data reinforce the biological plausibility of the SNPs in the OA. Second, our association study was limited to one population, so more studies in different populations are needed to support our findings, as well as to evaluate the functionality of the associated SNPs and be able to show evidence of whether they have a causal effect or not. Finally, there are more variants of the same gene that were not analyzed, as well as other genes of the ECM that were not considered and whose impact on OA development is unknown. Therefore, these preliminary results must be interpreted with caution, regarding our observation of a non-association between some polymorphisms and knee OA.

In summary, we evaluated nine polymorphisms associated with components of the articular cartilage ECM in knee OA patients in a Mexican population using the MDR and GMDR methods. The rs679620 (*MMP3*), rs699947 (*VEGFA*), and rs 1800255 (*COL3A1*) polymorphisms showed epistasis. Knowing the interactions involved in ECM metabolism could provide a new tool to identify individuals at high risk of developing knee OA. Further studies are needed to establish the mechanisms of interaction between these polymorphisms and their effects on knee OA susceptibility. This work highlights the importance to open the possibility of evaluating other pathways involved in ECM metabolism, such as the bone morphogenetic protein (BMP), fibroblast growth factor (FGF), hypoxia-inducible factor (HIF), nuclear factor kappa-B (NF-κB), mitogen-activated protein kinase (MAPK), and hedgehog (Hh) signaling pathways.

## Supplementary material

The following online material is available for this article:

Table S1 Ancestry informative markers (AIMs) studied.

Table S2 Polymorphisms distribution in groups stratified by gender and age groups.

Table S3 Polymorphism interactions by logistic regression.
