# Supplementary material for: Epistasis of polymorphisms related to the articular cartilage extracellular matrix in knee osteoarthritis: Analysis-based multifactor dimensionality reduction
Source: Genet Mol Biol. 2020 Mar 27;43(2):e20180349. doi: 10.1590/1678-4685-GMB-2018-0349 (PMC7197998; doi:10.1590/1678-4685-GMB-2018-0349)
Supplement: Supplementary file 1 [file 1415-4757-GMB-43-2-e20180349-suppl1.pdf]

Supplementary Material to "Epistasis of polymorphisms related to the articular cartilage extracellular matrix in knee osteoarthritis: Analysis-based multifactor dimensionality reduction"

Table S1 - Ancestry informative markers (AIMs) studied.

AIMs	db SNP rs ID	Chromosome: position	Location	MAF in Mexican population
DRD2	rs1800498	11:113291588	Intron	0.33 (A)
-	rs2862	15:35145553	5'UTR	0.59 (C)
-	rs223830	16:57451971	3'UTR	0.40 (C)
CA10	rs203096	17:50011769	Intron	0.50 (T)
CKM	rs4884	19:45810035	-	0.58 (A)
PRKCE	rs281478	2:46400411	5'UTR	0.06 (C)
-	rs722098	21:16685598	Intergenic	0.58 (A)
SAP30L	rs3340	5:153831867	Intron	0.48 (C)
-	rs2695	9:82884577	Intergenic	0.60 (T)
