# Supplementary material for: Epistasis of polymorphisms related to the articular cartilage extracellular matrix in knee osteoarthritis: Analysis-based multifactor dimensionality reduction
Source: Genet Mol Biol. 2020 Mar 27;43(2):e20180349. doi: 10.1590/1678-4685-GMB-2018-0349 (PMC7197998; doi:10.1590/1678-4685-GMB-2018-0349)
Supplement: Supplementary file 2 [file 1415-4757-GMB-43-2-e20180349-suppl2.pdf]

Supplementary Material to "Epistasis of polymorphisms related to the articular cartilage extracellular matrix in knee osteoarthritis: Analysis-based multifactor dimensionality reduction"

Table S2 - Polymorphisms distribution in groups stratified by gender and age groups.

	**Female**					**Male**				
**Gene (SNP rs ID)**	**OA N (%)**	**Controls N (%)**	**OR**	**(95% CI)**	***p***	**OA N (%)**	**Controls N (%)**	**OR**	**(95% CI)**	***p***
COL3A1 (rs1800255)										
GG	29 (39.2)	28 (46.7)	1.00	Reference		6 (60.0)	2 (11.1)	1.00	Reference	
GA	38 (51.3)	30 (50.0)	2.21	(0.94 – 5.18)	0.06	4 (40.0)	15 (83.3)	**0.07**	(0.00 – 0.84)	**0.03**
AA	7 (9.5)	2 (3.3)	3.29	(0.53 – 20.2)	0.19	0 (0.0)	1 (5.6)	-	-	-
G	96 (64.9)	86 (71.7)	1.00	Reference		16 (80.0)	19 (52.8)	1.00	Reference	
A	52 (35.1)	34 (28.3)	1.71	(0.95 – 3.09)	0.07	4 (20.0)	17 (47.2)	0.28	(0.06 – 1.25)	0.09
VEGFA (rs699947)										
CC	31 (44.3)	39 (48.1)	1.00	Reference		8 (72.7)	18 (54.5)	1.00	Reference	
CA	32 (45.7)	29 (35.8)	1.64	(0.72 – 3.73)	0.23	2 (18.2)	12 (36.4)	**0.04**	(0.00 – 0.85)	**0.03**
AA	7 (10.0)	13 (16.1)	0.63	(0.18 – 2.13)	0.46	1 (9.1)	3 (9.1)	0.19	(0.00 – 12.6)	0.44
C	94 (67.1)	107 (66.0)	1.00	Reference		18 (81.8)	48 (72.7)	1.00	Reference	
A	46 (32.9)	55 (34.0)	0.96	(0.55 – 1.67)	0.90	4 (18.2)	18 (27.3)	0.26	(0.05 – 1.29)	0.10
EGF (rs4444903)										
GG	18 (25.4)	22 (27.9)	1.00	Reference		5 (45.4)	9 (28.1)	1.00	Reference	
GA	39 (54.9)	38 (48.1)	1.58	(0.66 – 3.79)	0.29	2 (18.2)	18 (56.3)	**0.10**	(0.01 – 0.94)	**0.04**
AA	14 (19.7)	19 (24.0)	1.52	(0.51 – 4.48)	0.44	4 (36.4)	5 (15.6)	2.43	(0.21 – 27.4)	0.47
G	75 (52.8)	82 (51.9)	1.00	Reference		12 (54.6)	36 (56.2)	1.00	Reference	
A	67 (47.2)	76 (48.1)	1.23	(0.73 – 2.08)	0.41	10 (45.4)	28 (43.8)	0.92	(0.29 – 2.93)	0.89

	**≤50 years**					**>50 years**				
**Gene (SNP rs ID)**	**OA N (%)**	**Controls N (%)**	**OR**	**(95% CI)**	***p***	**OA N (%)**	**Controls N (%)**	**OR**	**(95% CI)**	***p***
MMP3 (rs679620)										
CC	22 (44.9)	33 (51.6)	1.00	Reference		17 (54.8)	8 (42.1)	1.00	Reference	
CT	17 (34.7)	10 (15.6)	**3.09**	(1.07 – 8.93)	**0.03**	13 (41.9)	7 (36.8)	0.83	(0.15 – 4.39)	0.82
TT	10 (20.4)	21 (32.8)	1.21	(0.41 – 3.54)	0.71	1 (3.3)	4 (21.1)	0.08	(0.00 – 1.27)	0.07
C	61 (62.2)	76 (59.4)	1.00	Reference		47 (75.8)	23 (60.5)	1.00	Reference	
T	37 (37.8)	52 (40.6)	1.31	(0.70 – 2.43)	0.39	15 (24.2)	15 (39.5)	0.43	(0.14 – 1.25)	0.12
VEGFA (rs699947)										
CC	25 (49.0)	47 (54.0)	1.00	Reference		14 (46.7)	10 (37.0)	1.00	Reference	
CA	20 (39.2)	31 (35.6)	1.17	(0.51 – 2.67)	0.70	14 (46.7)	10 (37.0)	0.79	(0.16 – 3.91)	0.78
AA	6 (11.8)	9 (10.4)	1.13	(0.31 – 4.16)	0.84	2 (6.6)	7 (26.0)	**0.06**	(0.00 – 0.86)	**0.03**
C	70 (68.6)	125 (71.8)	1.00	Reference		42 (70.0)	30 (55.6)	1.00	Reference	
A	32 (31.4)	49 (28.2)	1.10	(0.61 – 1.99)	0.73	18 (30.0)	24 (44.4)	0.40	(0.14 – 1.11)	0.08
HIF1AN (rs11292)										
AA	38 (80.8)	34 (54.9)	1.00	Reference		23 (82.1)	17 (85.0)	1.00	Reference	
AG	0 (0.0)	11 (17.7)	-	-	-	0 (0.0)	2 (10.0)	-	-	-
GG	9 (19.2)	17 (27.4)	0.49	(0.17 – 1.41)	0.19	5 (17.9)	1 (5.0)	3.44	(0.11 – 102.0)	0.47
A	76 (80.8)	79 (63.7)	1.00	Reference		46 (82.1)	36 (90.0)	1.00	Reference	
G	18 (19.2)	45 (36.3)	**0.44**	(0.22 – 0.88)	**0.02**	10 (17.9)	4 (10.0)	1.33	(0.22 – 8.05)	0.75

OA, osteoarthritis; OR, Odds ratio; CI, confidence interval. Significant *p*-values are in bold.
