# Supplementary material for: Epistasis of polymorphisms related to the articular cartilage extracellular matrix in knee osteoarthritis: Analysis-based multifactor dimensionality reduction
Source: Genet Mol Biol. 2020 Mar 27;43(2):e20180349. doi: 10.1590/1678-4685-GMB-2018-0349 (PMC7197998; doi:10.1590/1678-4685-GMB-2018-0349)
Supplement: Supplementary file 3 [file 1415-4757-GMB-43-2-e20180349-suppl3.pdf]

Supplementary Material to "Epistasis of polymorphisms related to the articular cartilage extracellular matrix in knee osteoarthritis: Analysis-based multifactor dimensionality reduction"

Table S3 - Polymorphism interactions by logistic regression.

Interactive polymorphisms		OR	95%CI	*p*
MMP3 rs679620	HIF1AN rs11292	1.04	0.62 – 1.74	0.94
	COL3A1 rs1800255	1.25	0.62 – 2.50	0.43
	VEGFA rs699947	1.16	0.23 – 1.64	0.33
	EGF rs4444903	1.39	0.81 – 2.40	0.24
HIF1AN rs11292	COL3A1 rs1800255	0.48	0.22 – 1.05	0.06
	VEGFA rs699947	0.77	0.28 – 2.11	0.61
	EGF rs4444903	0.96	0.19 – 2.37	0.53
COL3A1 rs1800255	VEGFA rs699947	0.89	0.24 – 3.38	0.86
	EGF rs4444903	1.33	0.28 – 6.33	0.72
VEGFA rs699947	EGF rs4444903	1.09	0.33 – 3.68	0.88
